# Understanding the biology of species' ranges: when and how does evolution change the rules of ecological engagement?

**DOI:** 10.1098/rstb.2021.0027

**Published:** 2022-04-11

**Authors:** Jon Bridle, Ary Hoffmann

**Affiliations:** ^1^ Department of Genetics, Evolution and Environment, University College London, London, UK; ^2^ School of BioSciences, Bio21 Institute, The University of Melbourne, Melbourne, Australia

**Keywords:** species' borders, range limits, evolutionary constraints, gene flow

## Abstract

Understanding processes that limit species' ranges has been a core issue in ecology and evolutionary biology for many decades, and has become increasingly important given the need to predict the responses of biological communities to rapid environmental change. However, we still have a poor understanding of evolution at range limits and its capacity to change the ecological ‘rules of engagement’ that define these communities, as well as the time frame over which this occurs. Here we link papers in the current volume to some key concepts involved in the interactions between evolutionary and ecological processes at species' margins. In particular, we separate hypotheses about species’ margins that focus on hard evolutionary limits, which determine how genotypes interact with their environment, from those concerned with soft evolutionary limits, which determine where and when local adaptation can persist in space and time. We show how theoretical models and empirical studies highlight conditions under which gene flow can expand local limits as well as contain them. In doing so, we emphasize the complex interplay between selection, demography and population structure throughout a species' geographical and ecological range that determines its persistence in biological communities. However, despite some impressively detailed studies on range limits, particularly in invertebrates and plants, few generalizations have emerged that can predict evolutionary responses at ecological margins. We outline some directions for future work such as considering the impact of structural genetic variants and metapopulation structure on limits, and the interaction between range limits and the evolution of mating systems and non-random dispersal.

This article is part of the theme issue ‘Species’ ranges in the face of changing environments (Part II)’.

## 1. The importance of range limits

Scientists have tried to understand the biological processes that create geographical range limits even before the disciplines of ecology and evolution existed [1]. The width and position of species’ geographical distributions depends on when and where populations persist in response to changing conditions in space and time, rather than becoming extinct. Tackling this issue requires consideration of population genetics and population ecology, of the ability of a given genotype to create different phenotypes, and of the impact of abiotic factors and species' interactions on the dynamics of populations, particularly at margins. The factors that determine range limits therefore also shape the diverse life histories and environmental sensitivities of phenotypes within populations, as well as the composition, diversity, resilience and productivity of the ecosystems that these populations create. Explaining range limits is also central to understanding gradients in diversity across ecosystems and latitudes, both within and between species. For instance, why do adaptive radiations produce many species with apparently narrow niches on oceanic islands and in the tropics, while local adaptation within fewer and more broadly distributed species seems more common in temperate seas and at higher latitudes?

There is increased urgency to understanding distributional limits because coming decades will be marked by rapidly changing and novel conditions, with communities fundamentally altered by habitat destruction, invasive species, pollution events and climate change [2]. Climate change in particular will (on average) make environments warmer and more variable as well as less predictable, making the evolution of novel traits and phenologies that minimize the impact of these new regimes necessary [3]. Although some species will successfully invade new ecological communities (as exemplified by invasive pest species, host switches and epidemics), the distribution of many species is likely to contract as the habitats they currently occupy (and the strategies they use to inhabit them) become increasingly unsuitable. As such species are lost from communities, those that depend on them will also become more prone to local extinction, reducing the complexity of ecosystems, as well as their resilience to future environmental change.

Despite the urgency of such rapid environmental change, we still struggle to predict which species will contract, maintain, or expand their distributions, and where (and when) in their geographical ranges this will happen, even though we see more and more examples of each of these changes across taxa and biomes. In addition, models that predict the impact of climate change on species and communities rarely consider many ecological and most evolutionary processes, precisely because their likelihood and impacts are largely unknown [4–6]. Instead, predictions are commonly based on species' distribution models (SDMs), where the current range of a species is associated with a suite of climatic factors that defines its niche. The future position and size of a species’ range is then determined by where these climatic factors will prevail in the future [7–9]. However the SDM approach is likely to be inadequate in many instances [10]. For many organisms, this will be because climate models rarely predict future (or current) environmental variation at the spatial and temporal scale at which genotypes experience them. For example, microclimates can vary as much across a few metres or minutes as average climate variables vary across hundreds of kilometres or years [11,12]. SDMs may also lack predictive power because our understanding of existing distributions (and therefore species' ecological limits) is very sensitive to sampling intensity and duration. For example, detecting range contractions (and species’ absences) at low elevations or low latitudes requires much more intense and finer-scale sampling than detecting range expansions (e.g. [13]) leading to an ‘extinction debt’ problem [14], that risks underestimating the effects of future climate change by overestimating species' existing tolerances.

Beyond these practical difficulties in sampling, SDMs also may not capture biological factors that are fundamental to most species’ local and global distributions. For example, many do not consider interactions between species [15] and how they tradeoff with climate variation [16], or how phenological and other types of plasticity [17] and non-random dispersal [18] affect the exposure of genotypes to climatic conditions at a given locality, or their functional role [3].

Other factors also affect the quality of predictions based on SDMs to different extents in different species, including historical factors [19], local adaptation across the existing range [20] and any novel adaptation in the invasive range [21]. In contrast to issues of sampling biases or intensity, the effect of adaptive divergence within species on their distributions depends on evolutionary rates, trajectories and limits [22]. These processes influence variation in ecological and life-history traits within and among populations, and therefore the patterns of gamete and zygote dispersal and species' interactions that shape biological communities, as well as the capacity for future evolution within species.

To understand range limits, a key issue therefore is to understand under what circumstances (and by how much) evolutionary rates and trajectories [22] determine the ecological ‘rules of engagement' that shape species’ niches, and therefore their distributions [23]. In the papers in this volume, many hypotheses and data relating to the evolution and ecology of range limits and range expansions are revisited and viewed from new perspectives. These papers highlight studies that emphasize the dynamic nature of species' ranges and how they relate to past and present evolutionary and ecological interactions. They reveal how species’ life histories, phylogenies and biotic interactions determine range limits, and they stress the importance of considering these patterns across sufficient time frames, as well as appropriate spatial scales. They also highlight the promise of new empirical tools and increasingly sophisticated theoretical models, even if many of the critical parameters of the models (such as genetic variation in fitness and its effect on population dynamics) remain difficult to measure. These papers also demonstrate a growing appreciation for the complexity of factors affecting range changes, the substantial differences between even closely related species in how these factors interact, the importance of understanding population ecology and genetics across the entire species' ranges (not just at population margins), and the role of past adaptation in determining ongoing responses to environmental change.

## 2. Understanding the evolution of ecological limits

Ecological and evolutionary explanations for species’ range limits date back to Darwin [1] who argued that interactions between abiotic factors and interspecific competition determine niche width as part of his analogy of a ‘tangled bank’. Darwin also argued that biotic interactions should increase at low latitudes, with abiotic effects on fitness dominating at higher latitudes, something for which there is now compelling empirical support [24]. The importance of biotic interactions in determining range limits remains an important theme in both modelling [25] and empirical [15,26] studies. In addition, adaptive divergence within species that depends on interactions between spatially variable selection and gene flow has become central to explaining range limits, based on insights from models that integrate population genetics with population ecology [27].

Over the past 50 years, reviews on the biology of species' margins (e.g. [28–31]) have given different weight to different evolutionary explanations as new types of data have become available, alongside the development of models for ecological margins, and an increased diversity of organisms being studied. For instance, although limits to genetic variation were often emphasized in early reviews [28,29], some reviews [30,31] highlighted the effects of gene flow more strongly, coinciding with the development of models stressing how gene flow swamped local adaptation rather than facilitating it [32,33]. However, later models and simulations [34,35] focused attention on the role of gene flow in increasing adaptive potential, while also reducing the power of selection relative to drift because of the effect of increased genetic variance on population mean fitness. Where this ‘genetic load’ of standing variation becomes sufficiently high, it prevents the trait mean from matching its local optimum, leading to maladaptation load. Where such trait mismatch occurs, or where variance increases abruptly (at a steepening gradient, for example), population density declines, leading to asymmetric gene flow from neighbouring (better adapted or with lower variance) populations, and causing range limits in space wherever the environmental gradient exceeds a threshold steepness. The increasing sophistication of such models integrates population ecology and genetics, focusing attention on measuring how trait optima change in space and time, and how variation in traits and fitness relates to population density, and to range margins in nature. In particular, perhaps owing to the hugely increased availability of genomic data, the most recent reviews of evolutionary limits again emphasize the need to consider genetic variation in key traits, which must always be limited in some directions (e.g. [36]).

**Figure 1.**
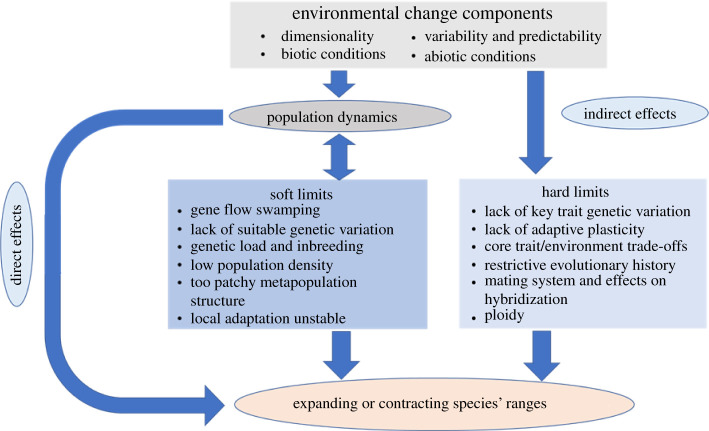
The interaction between environmental changes with processes that affect hard and soft evolutionary limits and their impact on species' ranges. Environmental conditions at margins can change through different components which influence population dynamics and/or determine whether hard limits are exceeded. Changes in population dynamics can have direct ecological effects on whether range margins expand/contract or whether species invade/are lost from a community. In addition, the conditions at margins and across a species' range exert indirect effects on those evolutionary processes influencing soft and hard limits. (Online version in colour.)

We think a useful way to explore different explanations for evolutionary limits to species' ranges is to divide them into (i) ‘hard’ and (ii) ‘soft’ limits, and place these within the environmental context under consideration (figure 1). Although this distinction is useful, these different kinds of limits are really at each end of a continuum characterized by the rarity of mutations that alter a genotype's fundamental relationship with its environment (hard limits) compared to those that affect its spread along some aspect of its existing environment (soft limits). In this sense, ‘hard limits' (as emphasized by Antonovics [28] and Hoffmann & Blows [29]) are those that will always restrict a species' distribution when populations are exposed to a given set of conditions, because they reflect inherent limits or trait thresholds that cannot be easily overcome, at least without the spread of novel or rare mutations that alter existing phenotypic trajectories or key developmental or ecological trade-offs. By contrast, we define ‘soft limits’ as those driven by population genetic processes such as gene flow and local adaptation that determine how already existing alleles are arranged in space or time. For example, where local adaptation (i.e. trait matching to the optimum) is common, a species will form a ‘long, thin’ distribution along a spatial environmental gradient, because its ecological niche is widened. However, this local adaptation also means that the standing genetic load created by gene flow between populations generates reduced but similar population densities everywhere, except at the margins. By contrast, where local adaptation is rare, a species' distribution will be ‘short and fat’ (i.e. with a narrow ecological niche) because most phenotypes only match the local optima within a small part of ecological space. However, population density remains high here (but only here) because gene flow and recombination does little to increase standing load. Once a species' distribution is characterized by the latter situation (e.g. a highly specialized, ‘patchy’ distribution), adaptation at the ecological margin becomes harder, because most productivity is only at the range centre, effectively devaluing evolution at the edge [37]. In this way, ‘soft’ limits are more fluid and dynamic than ‘hard’ limits in the way they respond strongly to local population ecology and gene flow, and how they affect trait variation and population density. At soft evolutionary limits therefore, the thresholds generated by feedback between local adaptation, density, and gene flow will sometimes cause sudden and substantial range expansions and contractions, even in response to slow, minor or local environmental changes.

The relative importance of hard and local soft evolutionary limits depends on where in ecological space populations are currently located. Broadly speaking, when all necessary phenotypes are accessible by selection on alleles already segregating across their range, species' distributions will be largely dominated by soft limits. By contrast, in regions of ecological space where the necessary phenotypes are less accessible (and demand the spread of novel mutations, or rare combinations of alleles), population persistence in space and time will largely be determined by hard limits.

**Figure 2.**
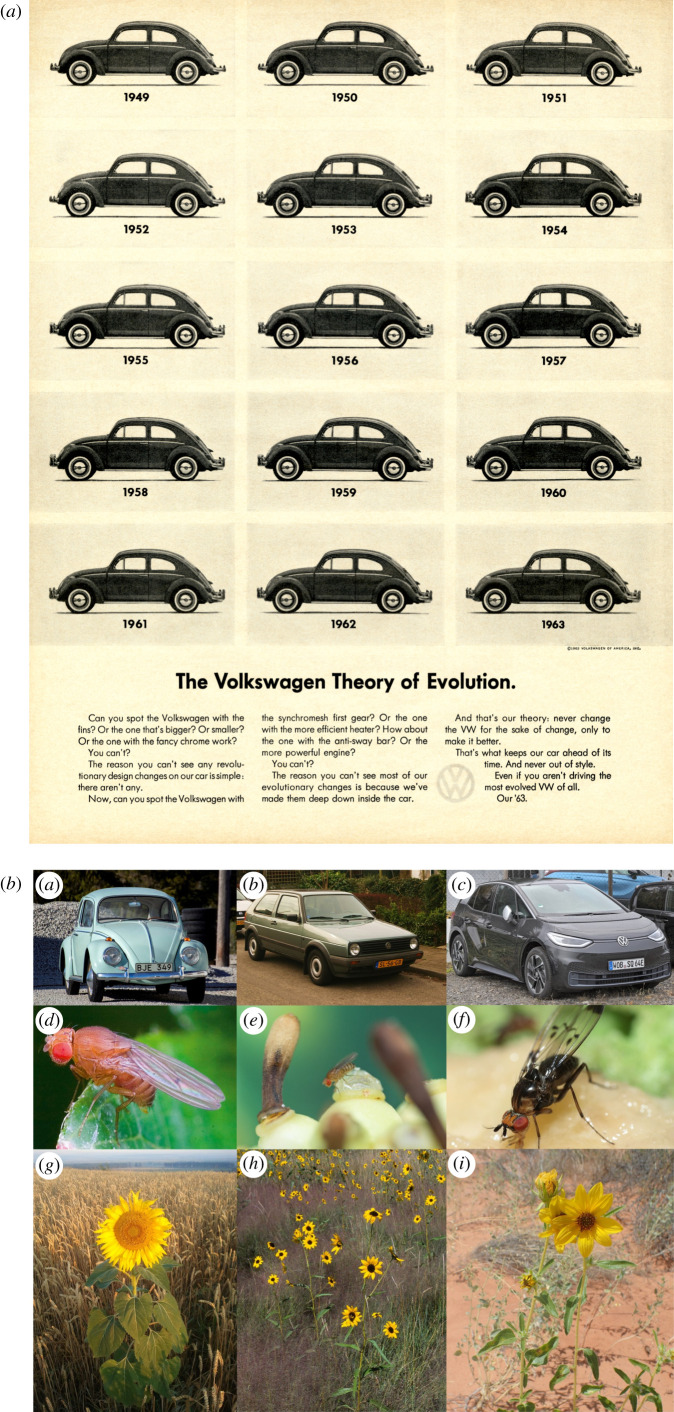
Overcoming evolutionary constraints in Volkswagen cars and biological systems. (*a*) An advertisement from Life-magazine in 1962, used with permission from Volkswagen Aktiengesellschaft. (*b*) Constraints to phenotypic organization (rear internal combustion engines) (*a,* Beetle model) act as a hard limit to the evolution of models with front engines that can provide other uses. However, such hard limits may hide substantial soft changes in interior design and internal organization (such as the move from carburettors to fuel injectors). Fundamental changes in organization were required to allow a frontal internal combustion engine (*b,* Golf or Rabbit model) and further changes to battery power (*c,* ID-3 model). These changes allowed soft adaptation within new (wider or narrower) ecological domains and in new directions, (such as as a future home power source in the case of the ID-3), perhaps allowing their spread into novel habitats. Biological species show similar hard constraints that limit their ecological ranges and that can be overcome through the development of core mutations (e.g. *e, Drosophila sechellia* has evolved novel odorant receptors to use *Morinda* fruit, compared to the generalist foods of its continental close relative, *D. simulans* (*d*); similarly (*f*) many Hawaiian *Drosophila* have evolved novel and specialised feeding or mating behaviours); or the introduction of multiple new alleles introduced through hybridization allowing for hard limits to be crossed, and the population genetic exploration of novel environmental domains in various (*g*,*h*,*i*) *Helianthus* species). (Image credits: (*a*) Bene Riobó/Wikimedia Commons (CC BY-SA 4.0); (*b*) Niels de Wit/Wikimedia Commons (CC BY-SA 2.0); (*c*) Alexander Migl/Wikimedia Commons (CC BY-SA 4.0); (*d*) Dr Andrew Weeks/Wikimedia Commons (CC BY-SA 3.0); (*e*) Benjamin Fabian; (*f*) KarlM/Wikimedia Commons (CC BY-SA 3.0); (*g*) Martinas Angel/Wikimedia Commons (CC BY-SA 3.0); (*h*) Photograph by Robert Sivinski; (*i*) Kenraiz/Wikimedia Commons (CC BY-SA 4.0).) (Online version in colour.)

An analogy that might help in understanding our distinction between hard and soft evolutionary limits is in the design of cars; we use an urban model from Volkswagen as an example. The basic shape of the original ‘Beetle’ design presented a hard limit to evolution associated with a rear-mounted air-cooled engine, albeit one within which a lot of local (soft) adaptation could occur by modification within these limits using existing components (e.g. in engine transmission, engine size and interior design), despite the Beetle's outward appearance (and the largely urban limits of its distribution) remaining famously unchanged (figure 2*a*). However, in the 1970s, a ‘hard’ evolutionary shift to a front-mounted liquid-cooled engine resulted in a different body shape as typified by the ‘Golf’ (or ‘Rabbit’ in the USA; figure 2*b*) which then led to further (soft) rearrangement of available standing variation, again within the hard evolutionary constraints of this new body shape. The current move to electric vehicles has established a new hard evolutionary limit as represented by the ‘ID.3’ (figure 2*c*) where batteries are positioned low alongside other changes to its body plan allowing increased interior and luggage space, and soft adaptation within different parts of ecological space. This new relationship with the environment is allowing rapid ‘soft’ evolution through the increased versatility such a layout affords, which may permit future expansion of this originally urban design along the steep selective gradients that mark the transition to suburban habitats, increasing the range size of the ID.3 relative to its ancestors. The fundamentally distinct (hard) evolutionary trajectories that these three designs explore are modified as these vehicles expand their distribution by invading different urban habitats around the world for which they are at least partially pre-adapted, albeit with adaptation at (soft) limits restricted by local factors such as dispersal and interactions with national governments, and (we hope) increasing awareness of the folly of private car use in urban environments (i.e. the evolution of new biotic interactions).

## 3. Hard evolutionary limits and the traits that define them

To break hard evolutionary limits, mutations that have large impacts on developmental processes or that lead to novel metabolic pathways and physiological/behavioural processes may be required (as in the evolution of olfactory receptors in *Drosophila sechellia* to use *Morinda* fruit (figure 2*e*, [38]) or the evolution of specialized feeding and sexual behaviours in Hawaiian *Drosophila* (figure 2*f*, [39])) unless there is a sudden influx of many novel genes affecting phenology, physiology and life history through hybridization as in sunflowers (figure 2*g–i*, [40]). Although genetic variation may still exist for individual traits, further adaptive divergence in these traits may be limited by trade-offs that cannot be overcome without substantial and fundamental changes in genomic organization or life history. Genetic variation might also still be present for traits but hidden from selection owing to condition dependence or plasticity, often perhaps at the points in ecological space where the variation is most needed (e.g. [41]). Signatures of hard limits may include low levels of heritable variation in field populations for critical traits limiting ecological tolerances (as has been shown in some *Drosophila* for aridity and high temperature [42]), or an inability for even strong selection to change trait means that have some heritable variation [43]. Species' limits in such cases should also relate to the design and physiological limits inherent in how phenotypes operate [44]. Understanding hard limits therefore requires a greater focus on the phylogenetic and development processes that determine trait correlations and available genetic variation than understanding soft limits.

However, there is a continuum between soft and hard limits because low evolvability in key traits could also be driven by: (i) a small population size exhausting genetic variation generally (a soft limit); (ii) prevailing conditions at a particular location creating ecological trade-offs and/or genomic associations with other fitness traits [45]); or (iii) the multidimensionality of fitness changes required to match the local trait optima [28,29] (a hard limit—at least until the trait optima shift). Some of these limits (e.g. (i)) could be overcome by gene flow along ecological gradients. However, gene flow is likely to increase relative fitness in the required evolutionary trajectories by a smaller amount as hard limits are approached ((ii) and (iii)), given much of the genotypic variance created will not be useful for local adaptation. Nevertheless, this non-adaptive phenotypic variance will still reduce population mean fitness and population density, making local extinction (i.e. a range margin) more likely in regions of ecological space where evolutionary limits are associated with trait correlations.

## 4. Soft evolutionary limits and the effects of gene flow on local adaptation

Much of the emphasis in understanding soft limits has been on how gene flow among populations prevents or effects local adaptation, something that has been considered extensively in theory [32,33,35,46], although empirical tests have proved difficult and labour-intensive. One challenge is that gene flow is often as much driven by ecological factors, and by local fragmentation of habitats, as by larger-scale geographical patterns [47], meaning that directional gene flow towards species’ ecological margins cannot be assumed. Instead, local effects on population density are more likely to determine the magnitude and direction of gene flow, and where gene flow creates abrupt ecological margins, compared to where it increases the geographical area from which potentially advantageous alleles can be harvested [37]. Presumably more (or more asymmetrical) gene flow is needed to prevent local adaptation, compared to the amount needed to supply the variance in relative fitness necessary for adaptive divergence at soft limits. However, we lack empirical tests of where (asymmetrical) gene flow is important in limiting niche width [48], compared to spreading favourable alleles, and even less about this interaction in natural populations, or how it varies across traits with different kinds of genetic architecture.

Population densities at soft range limits can also be widened by plastic responses if these improve the match of traits to local optima. However, plastic responses are likely to have costs, which may be direct in terms of production costs, or indirect where non-adaptive plastic responses are triggered by novel environmental conditions [3]. Within existing ecological limits, such adaptive plasticity may aid population establishment or persistence, but slow evolutionary responses by masking alleles that affect fitness, at least until the adaptive value of existing plasticity is exceeded [49]. Eriksson & Rafajlović [50] consider a case where genetic variants that allow plastic changes have costs, as well as modelling increases in genetic variance in plasticity owing to gene flow. They derive conditions where plastic changes at range limits should evolve, in relation to their cost, and to levels of temporal environmental variation. Similar predictions are now being tested empirically [51,52], and reveal reductions in mean population fitness owing to non-adaptive plasticity in novel environments, along with increases in relative fitness that provide the potential for rapid evolution in plasticity at ecological margins. However, detecting metabolic or sensory costs to plasticity remains difficult.

## 5. Evolutionary limits as illustrated by invasive species'

When an invasive species is expanding into a new environment, ecological processes alone can shape the expanding range of the species (e.g. dispersal, interactions with existing species, suitability of abiotic conditions) until some soft ecological limit is reached, possibly owing to interactions with resident species or abiotic factors. Local adaptation (predominantly using existing variation) may then be necessary for further expansion, meaning that population genetic processes (gene flow, local adaptation, genetic load) may become increasingly important in determining ecological margins over an invasion's history [53]. However, how these population processes interact will depend on the environment at this initial range limit, including its dimensionality, and environmental variability in space and time. In particular, acclimatization to environmental stress, as may be common during invasions, may reduce genetic variation in key traits, slowing adaptive potential at ecological margins, as suggested in salmon [54]. Biotic interactions probably present strongly multidimensional adaptive challenges to invading populations, particularly when acting in concert with abiotic changes [55]. Such combinations of biotic and abiotic interactions may create hard evolutionary limits, that arrest the spread of invasions for long periods, until they are overcome through novel (potentially very rare) mutations [56], or by hybridization with resident species, or polyploidy (see below). They may also be overcome if novel environmental conditions remove hard limits, such as in purple loosestrife (*Lythrum salicaria*)*,* where the fitness consequences of a core trade-off between flowering time and reproductive output is altered in the invasive range of this species [57]. The fact that invasive organisms are often somewhat pre-adapted to their new environment (and show niche conservatism in terms of their ecological limits) may reflect the importance of hard evolutionary limits in determining the composition of ecological communities [58], even if adaptation to soft evolutionary limits is possible after invasion has occurred [59].

## 6. Identifying evolutionary limits determined by genetic variation in traits

Working out whether a given trait is at a hard evolutionary limit is challenging. For a start, the relevant trait(s) needs to be identified and measured. This challenge is typified by thermal limits—the extent to which species can tolerate extremes of high and low temperature. Although simple conceptually, thermal limits can be measured in many contrasting ways and need to be considered within an often complex environmental context [60]. In addition, thermal limits can be plastic, as highlighted by Noer *et al.* [61], who show how the ability of insects to plastically increase their heat tolerance owing to local weather conditions varies among insects collected from the field, hardly changing in some species but more than doubling in others. Although this variability has been noted in laboratory studies on related insects, the Noer *et al.* study highlights particularly high levels of variability under field conditions, presumably linked to field microclimate variation [62] as well as variation in condition and genotype. These results mean that thermal limits generated by a combination of genetics and plasticity are very difficult to measure, especially when considered in a field context.

Even once relevant traits have been identified, empirical tests of genetic variation in these traits (and other interacting traits) remain difficult. This is particularly true under field conditions and at ecological margins where target species are (by definition) at low density, making them difficult to collect and phenotype in large numbers. Understandably few studies have taken such a heroic research direction [36,63]. However, they provide some examples where rapid evolution (and range expansion) at margins reduces standing variation in ecologically relevant traits, which may potentially compromise future adaptive shifts (e.g. [64,65]). It is usually unclear whether a reduction of genetic variation associated with rapid evolutionary responses represents a new hard evolutionary limit at the new ecological margin, or just a delay before standing variation is replenished (i.e. recreating a soft limit). In the absence of genetic data on specific traits, transplant experiments beyond ecological margins can test whether conditions are suitable beyond them [66], by indirectly testing whether lack of genetic variation limits range expansion. However, if extreme events determine species' distributions, resulting in long lag times before even local extinctions occur, experimental tests using transplants need to be conducted over many generations [67].

In addition, trade-offs between traits are hard to assess, and it is even harder to work out whether these represent hard limits (e.g. caused by genomic organization) or soft limits that will change as allele and genotype frequencies change owing to local selection, recombination and genetic drift, and as phenotypes are affected by local environments. This hypothesis has been considered empirically in taxa such as *Drosophila* that can be measured easily [68], and in plants that can be transplanted and measured within and across generations [69] as well as in mammals and fishes [41,52,70]. For example as mentioned above, the northern limit of the purple loosestrife (*L. salicaria*) [57] is set by a trade-off between size at, and time to, flowering. This is likely to represent a hard evolutionary limit that demands substantial life-history evolution to overcome, despite rapid adaptation to climate elsewhere in its range. In the case of *Drosophila serrata*, selection for increased cold resistance was repeatedly associated with a decrease in fecundity [68]. However, as in the case of heritable variation, it is difficult to estimate antagonistic interactions and how they limit the increases in relative fitness expected owing to gene flow. It is even more challenging to demonstrate the effect of directional selection on key traits in the wild, and their effect on adaptive potential [71].

Although genetic variation in plants can be relatively easily measured in the field through common garden experiments and transplants [51,66], this is much more challenging in mobile animals where laboratory experiments are often used instead, and where any field experiments usually demand restrictions to the mobility of genotypes, making measurement of their natural exposure to environmental variation difficult. Even in plants, transplant experiments that use cuttings to estimate fitness across environments within genotypes need complementary tests of pollen or seed movement to reflect how alleles typically move in natural populations, and to incorporate maternal or early-life effects on phenotypes. O'Brien *et al.* [26] demonstrate that laboratory-measured variation in fitness in *Drosophila* rarely relates to field performance, particularly when measured along natural ecological gradients that vary in time as well as space. Their studies also highlight how available genetic variation in the field (as well as its fitness consequences) can be highly environment-dependent, and may act in concert with environmental variation to limit local adaptation in space and time. Such environmental limits to genetic variation may be particularly common if interactions between species dominate fitness, for example where species replace each other along abiotic gradients (i.e. their ecological margins) [72].

Models by Alexander *et al.* [55] support the idea that trade-offs with biotic factors in particular decrease the evolutionary potential of populations at range limits, especially limits that are determined by climate. Also, Stewart *et al.* [73] consider the butterfly *Aricia agestis* that has undergone a host shift that has reduced its host plant repertoire at its UK range margin. This host shift has allowed its spread to warming sites further north, probably through the evolution of novel genotypes at the range edge, and the loss of ancestral polyphagy. However, the value of the host plant it now uses exclusively for larval growth depends on a narrow window of climate conditions in early spring, making periodic range contractions and subsequent expansions likely. This means that switching to monophagy (although apparently favoured by selection during increasingly common warm years) may become an unreliable strategy in the more unpredictable environments of the future, decreasing or fragmenting the species' geographical range [64].

## 7. Comparative studies and hard range limits

Given the difficulties of linking low evolvability of traits and trait interactions to hard ecological limits across populations in space and time, comparative studies across evolutionary time might be more useful in identifying such limits. Willi & Van Buskirk [74] provide a review of trade-offs involving populations of species at temperature extremes and dry conditions, and highlight the pervasiveness of the classic trade-off associated with completing growth versus reproduction when favourable conditions are short. Another long-standing issue considered through comparative analysis in this volume [75] is whether species with wide distributions have broader niche breadths, and how such an association is produced by a combination of their ecology and life history, and evolution across their ranges. Alternatively, range expansions may depend on bouts of speciation in lineages as documented in some bird groups [76] where speciation is apparently required to break hard evolutionary limits, creating daughter species with similar (or even narrower) niche widths than their parents. Overcoming one hard evolutionary limit may make overcoming a different one harder, confining species that do so to narrower evolutionary trajectories than their parent species (creating adaptive radiations into high levels of specialization, as often observed on oceanic islands; see figure 2*e,f*). Alternatively, once speciation has occurred, species' ages can quickly become uncorrelated with range size as noted for damselflies [77]. In this case, ecological limits may instead be determined by local adaptation *after* speciation, suggesting isolation from parental gene flow was necessary for rapid adaptive divergence along new phenotypic trajectories once hard evolutionary limits were overcome. However, testing such hypotheses is difficult, given it remains unclear to what extent current species' distributions correlate with their environmental tolerances. For example, Maccagni & Willi [75] conducted common garden experiments across 90 Brassicaceae species with different elevation ranges, and showed that high elevation species exposed to a wider range of temperatures are not necessarily more tolerant or more plastic when exposed to different levels of frost and heat. Similarly, mid elevation species with the widest elevation range, and exposed to the highest level of spatial thermal variability, were not necessarily the most broadly tolerant of environmental variation.

These studies suggest that historical as well as current processes need to be considered when comparing range size and niche breadth. Lancaster [78] investigates the ability of spatial eco-evolutionary processes to generate range size-niche breadth associations, by shaping latitudinal gradients during range expansion towards poles. She argues that, while local selection under equilibrium conditions should typically determine trait patterns across latitudinal gradients, this downplays the importance of spatial selection during range expansions in driving these changes. Historical patterns and the genetic and ecological effects of past adaptation may therefore be very important in determining species’ responses to future change [6]. Such studies also highlight the risk of assuming that existing populations are well-adapted at their current locations, a point reviewed in [79].

## 8. The evolution of gametic and allelic associations and ploidy at range margins

Three processes that can suddenly alter hard limits to species' distributions are changes in mating strategy, recombination and polyploidization. Although the loss of sexual reproduction could decrease the supply of favourable alleles through recombination, it may evolve at ecological margins because it frees organisms from previously specialized biotic interactions (e.g. with particular animals for plant pollination or seed dispersal) that may be limiting their expansion into new environments. However, Dawson-Glass & Hargreaves [80] provide only limited support for this hypothesis, suggesting that pollen limitation affects reproductive output in only a subset of plant examples. Again, such studies highlight the many patterns and processes associated with range limits, as well as their multiple and interacting effects. For example, increased selfing at a range margin could reduce genetic load and increase population growth rates (so increasing adaptive potential), even though it reduces the ecological and geographical area from which (potentially pre-adapted) genetic variation can be sourced. This could include access to alleles from other species through hybridization [38].

**Figure 3.**
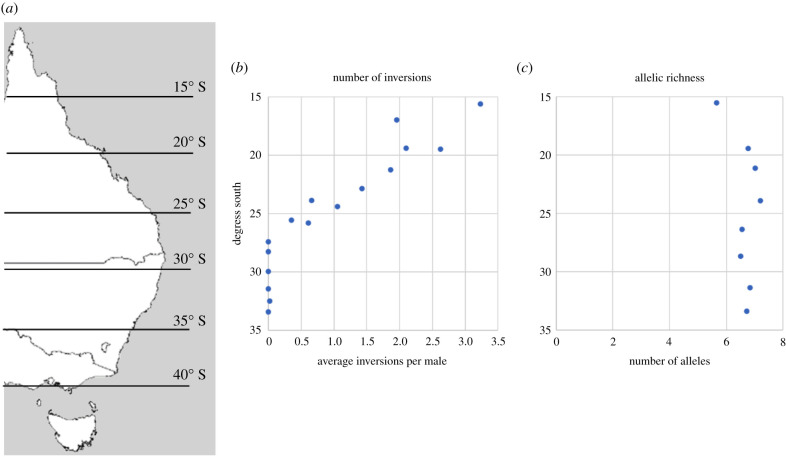
Survey map of eastern Australia (*a*) and patterns of (*b*) inversion frequency and (*c*) allelic richness (microsatellite variation) in *Drosophila serrata* from the northern part of the species' range (top) which extends into Papua New Guinea to the species' margin in the south of the range (right-hand side, at around 34° S). The species is distributed along the eastern coast of mainland Australia. Although allelic richness outside inversions is not affected much by proximity to the southern border, inversion polymorphism declines sharply, possibly because greater environmental variation at these margins demands greater recombination in genomic regions that are typically polymorphic for inversions elsewhere. Plotted using data from Stocker *et al*. [85] and Van Heerwaarden *et al.* [86]. (Online version in colour.)

The evolution of recombination was considered critical in early work comparing marginal and central populations across species (particularly in *Drosophila*). This emphasized the genetic differences between populations from the centre versus the edge of the species’ distribution involving overall declines in polymorphism as measured by inversion frequencies [81] (figure 3*b*). These reduced inversion frequencies result in a greater production of recombinants at the margins even though levels of genetic variation at other loci remain similar (figure 3*c*). Several hypotheses were proposed to explain these recurring patterns across species, including selection favouring inversion heterozygotes in central populations, or that responses to temporally varying selection at margins are less effective where chromosomal rearrangements are polymorphic in populations. The latter effect would lead to increased recombination rates at margins but no changes in allelic variation (meaning that allozyme/microsatellite variation would not decline much, as is indeed seen in *D. serrata* in figure 3)*.* Such patterns and explanations contrast with the idea that inversions and other structural rearrangements increase in frequency at ecological margins to maintain adaptive combinations of alleles that can persist in the presence of gene flow, facilitating speciation in the presence of gene flow as seen in *Heliconius* [82,83]. The interaction between structural variants and varying selection and recombination pressures at ecological margins is clearly complex and demands further exploration (table 1) with some theoretical progress now being made [84].

**Table 1.** Some priorities for future empirical studies of the evolution of range limits.

area	issue
effect of allelic and genomic architecture on local adaptation	the effect of the presence of inversions on evolutionary responses should be considered experimentally in novel environments. Central to this issue is the scale at which gene flow inflates relative fitness (adaptive potential), compared to reducing mean fitness (load), and how this depends on genomic architecture
evolution of mating systems and ploidy at range edges	given the central importance of gene flow on evolution at margins, we recommend increased focus on the evolution of mating systems at range edges, and their impact on standing genetic variation and on niche width
genomic organization and recombination in marginal populations	polymorphisms for structural chromosomal rearrangements that decrease recombination should be assessed in marginal populations of taxa (other than *Drosophila*)
effects of mutualistic versus antagonistic species' interactions on ecological limits and range expansions	social evolution in newly tractable systems such as plant-fungal interactions, or microbiomes has profound effects on exposure of alleles to environmental variation. Although such mutualisms may create interactions that typically widen species' niches, the high specialization required may limit future evolution, and range expansion owing to climate change
research on metapopulations across temporal and spatial gradients	the re-establishment of local adaptation in metapopulation systems, following a global selection event could test how local processes shape range margins, and how this varies with population size, trait variation, and gradient steepness. Also important is to test the potentially contrasting effects of synchrony among populations on (i) patch persistence and (ii) local adaptation
phylogenetic and ecological causes of trade-offs	tests for phylogenetic constraints and their importance in determining trade-offs across ecological regimes allow tests of the effect of past selection in determining the phenotypes that recombination or mutation can produce over tens or thousands of generations
genetic basis of plasticity and its evolution in novel environments	preventing sudden population collapse in the novel environmental regimes created by climate change and biodiversity loss may depend on the rapid evolution of new forms of plasticity. Predicting such responses requires focus on the genetics of plasticity, and its adaptive potential outside as well as within existing ecological ranges
environmental variance versus environmental unpredictability	temporal variance is likely to limit adaptation at range limits. However, environmental variation is likely to be more limiting where it is more unpredictable, as is expected owing to climate change. Research should correlate niche breath of species exposed to environmental variability versus environmental unpredictability, and its effect on the evolutionary potential of their populations at margins

Thirdly, polyploidization may suddenly increase adaptive potential (as well as potentially overcoming trade-offs and hard evolutionary limits), providing populations can grow fast enough (potentially asexually) to offset the demographic load generated by the large increases in variance in fitness generated soon after increases in ploidy. Given the central importance of gene flow in determining species' margins, a deeper focus on the evolution of mating systems and ploidy at range edges, and their impact on standing genetic variation and niche width, is recommended [87] (table 1), particularly for hermaphroditic organisms where rates of selfing and sexuality can vary along a continuum. At the same time, however, reducing the rate or distance over which alleles can move across a genome may limit further niche expansion by denying genomes access to favoured allele combinations from other genomes or populations [88].

Finally, just as changes in mating system, recombination and ploidy affect the movement of alleles among genomes, the evolution of non-random dispersal (e.g. seed dispersal via animal vectors, or oviposition choice by insects), and of adaptive phenology and plasticity determines the exposure of genotypes to other genotypes, and across environments [3]. The effect of such departures from random (diffusion) models are now being considered in theoretical models (see below), as well as the effects of the evolution of plasticity (affecting genotype exposure) on ecological limits [50].

## 9. Soft evolutionary limits related to gene flow and population genetic structure

The role of gene flow in shaping local adaptation is crucial for understanding soft evolutionary limits, in terms of determining where and when existing genetic variation is arranged in such a way that trait means match local optima. Gene flow can increase or decrease adaptive potential by spreading alleles across a species’ range that make it easier or more difficult for populations to track local optima, and so maintain high densities (and symmetrical gene flow). Predicting range limits therefore depends on understanding how positive effects of gene flow on adaptive potential (increased relative fitness) interact with their associated reductions in mean fitness (through increased variance load), and (if load becomes sufficiently high) the displacement of the local trait mean from the optimum (maladaptation load). Where rates of gene flow are too high relative to the rate of change in trait mean required for a given spatial distance (gradient steepness), and growth rates at the optimum are too low to compensate for the demographic cost of variance load, local adaptation cannot track a changing trait optimum, and evolutionary limits form [27,37,46]. At these points in ecological space, species are likely to replace each other. These scenarios are considered in several theoretical papers in the current volume, while O'Brien *et al.* [26] summarize their attempts to test such models empirically in natural populations, and the importance of interactions among species in creating locally steep ecological gradients.

Polechová [89] considers gene flow across two-dimensional rather than one-dimensional space (but with an ecological gradient in only one dimension), and demonstrates how neighbourhood size (which determines the spread of advantageous alleles) increases faster than genetic drift in two-dimensional compared to one-dimensional models. These models suggest that gene flow across two dimensions makes the beneficial effects of intermediate gene flow into small populations more powerful than predicted, even where genetic drift has big effects on allele frequencies, at least provided environments change relatively smoothly in space.

Predicting how interactions between gene flow, selection and population ecology affect evolutionary responses becomes more complex as environments become increasingly patchy relative to gene flow. Sachdeva *et al.* [90] model an island population exposed to gene flow from a much larger mainland population, resulting in a decrease in population size on the island, owing to increased genetic load as a consequence of this gene flow. This arrangement contrasts with Polechová's model of populations in continuous space whose densities are determined solely by local adaptation. Instead, Sachdeva *et al*. [90] model discrete patches with inherently different mainland versus island population sizes, creating an inevitably asymmetric movement of alleles from the mainland to the island, even if trait means were to perfectly match the optima in both patches. Such inherently asymmetrical gene flow creates an unstable situation in the peripheral population owing to fluctuations in genetic load, as migration brings in mainland alleles that are deleterious, albeit alongside others that sometimes block the expression of the deleterious effects of these alleles.

Sachdeva *et al.*'s model [33] also extends genetic architecture beyond additive models, given that negative and positive effects of alleles (and therefore their effects on load and adaptive potential), are masked in some situations. Similar complications apply where the expression of alleles is environment-dependent, masking or increasing genetic load in some places but not others. In particular, where deleterious alleles are recessive in their effect (as in the case of inbreeding load), even very small amounts of migration from large mainland populations to small island populations can maintain high levels of standing load, despite continual purging by selection owing to their effects on relative fitness, as demonstrated in island populations of song sparrows [91].

The importance of temporal variation at range edges in driving eco-evolutionary dynamics is emphasized by Holt *et al.* [92]. They show that the extent to which species' ranges shrink or expand under temporal environmental changes is mediated by extinction risks as well as impacts on genetic variation. These are both processes affected by metapopulation structure, as demonstrated by empirical studies on bay checkerspot [93] and Glanville fritillary butterflies, and assays of trait variation in metapopulations [94], as well as in experiments that test how patchiness and population turnover affect local adaptation. The Holt *et al*. [92] models also show that intermediate levels of temporal environmental variation increase rates of local adaptation by reducing Allee effects, and maladaptive gene flow, compared to selection.

Empirical research on gene flow and range limits has focused on several areas, from characterizing patterns of genetic variation across species’ ranges, to experimental consequences of manipulating gene flow between populations, and transplants at ecological limits. As seen in *Drosophila*, nuclear genetic variation does not necessarily reduce towards species' margins (figure 3), although weak patterns of decreased variation have been found in some animals and plants, albeit with many exceptions [95] particularly in plant taxa [96]. Also, surveys often consider declines in genetic variation at global limits, rather than at local margins, where genetic variation and population density is most likely to be driven by gene flow and its twin-effects of increasing relative fitness and reducing mean fitness [26,34]. Temporal variation in selection and gene flow along ecological gradients may also explain why experimental transplants and tests of fitness in the field often fail to meet predictions based on spatial selection across gradients, as illustrated in seasonally-limited selection for cold resistance in *D. serrata* [68], and in transplant experiments that test how variation in ecologically important traits affects field fitness in *Drosophila birchii* along rainforest elevational gradients [26].

Studies of trait evolution, demography, and environmental variation in metapopulations, which inherently embrace a range of spatial and temporal scales, are well suited to investigate the evolutionary ecology of species’ persistence. In particular, new theory by Barton & Olusanya [97] suggests that adaptive responses are more likely when the entire metapopulation is affected by an environmental change in a similar way, whereas divergent selection among demes will slow adaptive responses, especially when the niches or patches vary in their relative size [98]. This is something that seems evident in the Glanville fritillary butterfly in Åland [94]. We think a key avenue for research is to explore the re-establishment of local adaptation in such metapopulation systems, particularly following a pervasive selection event, or along a critical spatial ecological gradient (table 1). In such metapopulations, the amount of temporal synchrony among populations (see [99] for a review) may increase ecological persistence at large spatial scales, but constrain local adaptation across the metapopulation, maintaining narrow ecological niches [37].

Antagonistic species' interactions can limit local evolutionary responses by reducing local population densities, bringing them closer to the demographic and drift thresholds predicted by Polechová & Barton [46]. With high level of temporal or spatial patchiness, such species’ interactions may be variable across short distances. Parmesan & Singer [93] review how mosaic patterns of selection within species' ranges in birds and insects create local limits (themselves sometimes exacerbated by local adaptation), that can be explained by interactions between population ecology, ecological trade-offs and available genetic variation.

## 10. From range expansions to predicting responses to climate change: new insights

Studies of genomic data during range expansions are increasingly used to track the invasion routes of organisms with expanding distributions, and to document the involvement of anthropogenic processes in facilitating these. Hudson *et al.* [100] provide an example in two marine species, highlighting the power of such an approach. Other studies demonstrate the value of genomic data not only in tracking invasions once organisms arrive in an area, but also in testing for ongoing selective sweeps and the likely origin of the mutations involved in any *in situ* adaptation, and in tracking the potential for adaptive shifts as range extensions occur [101]. Similarly, Jahnke & Jonsson [102] discuss linking genetic data on population differentiation with the effects of biophysical changes on gene flow or dispersal, while Holman *et al.* [103] use molecular tools to track range shifts across oceans in an invasive ascidian. Although genomics-based approaches are undoubtedly useful in understanding species’ responses to climate change [104,105], there remain challenges in predicting adaptive potential of populations across gradients from genomic data alone [106], especially where common alleles of small effect influence local range limits, or there are hard limits not reflected in allelic changes across genomes.

In the future, rather than evaluating adaptive potential to novel environments on a case-by case-basis, more generalized predictions for ecological limits could come from relating variation in gene networks and their modularity to range limits across phylogenies, in relation to ecological dimensionality, or the topology of ecological networks in the ecosystems through which range changes are occurring. Similarly, the population genetic considerations outlined above suggest that (most of the time) management should aim to maximize overall genetic variation in fitness (and population density), providing the best opportunity for selection to ‘find the phenotypes’ that bring populations closer to local trait optima, rather than trying to match specific alleles or trait variation to particular environments, something that depends on an understanding of fitness variation that we do not have even for our most studied species [22].

Because of climate change and the increased rates and distances of anthropogenic dispersal, more and more invasions and range expansions are being recorded. Although most range expansions result in a species' invading areas and outcompeting resident species, hybridization may also occur, with outcomes ranging from adaptive introgression that increases invasiveness [38,107], to a new stable hybrid zone developing between species that may become trapped in regions of environmental transition. Although hybrid zones are typically associated with sharp changes in ecological conditions, Gilbert *et al.* [108] show how underdominant alleles that spread during range expansions can result in stable hybrid zones that become disassociated from ecological change, as described by Barton & Hewitt [109]. Ongoing selection against the heterozygotes may then lead to parapatric speciation. Such studies reflect the importance of G × G interactions (epistasis) in determining how easily alleles with differing effects and frequencies move together through species' ranges to where they are at highest fitness, and how local selection and demography will determine where they form steep clines, potentially away from their global optima. Such questions were considered critical by some of the first population geneticists, such as Fisher [110] and Haldane [111], and are now becoming much more tractable.

Within existing ecological limits, evolutionary potential (i.e. at soft limits) to climate change should largely be determined by levels of heritable variation found throughout the geographical range. Prakash *et al*. [112] consider the case of red spruce tested in replicated common gardens across the latitudinal range of the species, demonstrating that growth and phenological responses have low to moderate heritabilities that could support *in situ* adaptation, albeit to varying extents across their range. However, while plastic changes in phenology were possible, they often had a maladaptive effect on growth, suggesting that existing genetic variation within populations may actually limit climate adaptation in red spruce. Such a finding highlights a growing concern: historical forms of environmental sensitivity (plasticity) may no longer be adaptive in the novel environmental regimes of coming decades. Instead, preventing sudden population collapse in the ‘new normal’ may depend on the rapid evolution of new forms of plasticity, as previously soft evolutionary limits become hard limits, in that they demand fundamentally new forms of environmental sensitivity. Such concerns should stimulate focus on the genetics of plasticity [113], and its adaptive potential outside as well as within existing ecological ranges [50,51].

Situations across a species' geographical range are often complex, especially when changing abiotic conditions are combined with changing interactions among species, and community composition and network structure. In particular, populations and communities within existing ranges are often at least as vulnerable to climate change as those at species’ edges, as is often reflected when genomic tests for local adaptation are conducted systematically across a range. For example, *Euphydryas editha bayensis* exists as a metapopulation within its range, wherever local selection drives local populations to maladaptive phenotypes, especially when the environment is uncertain, or is characterized by frequent extremes, as Parmesan & Singer [93] show. In this species, selection for early or late emergence resulted in populations vulnerable to climate change, while in another population, new selection pressures from predators led to the evolution of reduced thermal tolerance.

Climate change vulnerability will ultimately involve a combination of ‘hard’ and ‘soft’ evolutionary limits to species' margins. Determining their importance will require comparisons that span taxa and latitudes, and that include comparisons between ecosystems with distinct patterns of environmental exposure, gene flow and life histories—for example, between terrestrial, aquatic and marine systems. This volume represents a key step in this process, by generating contrasts between hard limits to distributions driven by constraints to available genetic variation, and the soft limits driven by interactions between gene flow and selection that determine when and where local adaptation can be maintained. To inform such a comparison, we need more studies that test for phylogenetic constraints and their importance in determining trade-offs, allowing tests of how past selection on genomes constrains the genotypes and phenotypes that are currently possible.

## 11. Conclusion: ways ahead for understanding range limits

The way that interactions between ecological and evolutionary processes are emphasized typically reflects the taxa in question, as well as the intellectual background of the investigating scientists. This makes understanding the biology of range margins a rich and rewarding field for collaborative research, driven by increasing commonalities between the ecosystems and taxa being studied, by the centripetal force of genomic analysis, and by increasingly complementary concerns and conclusions among researchers. We have highlighted several issues for further study throiughout this paper and summarized in table 1.

In particular, and thanks to improving genomic and ecological monitoring tools, and refined theoretical predictions, empirical research can now much better address two fundamental questions in range limit evolution: where in the parameter space explored by theoretical models do real ecological limits occur? Also, what does this tell us about which ecological and genetic parameters are most critical in structuring ecological communities and their function?

Although there are plenty of ecologically important traits suitable for investigation, more effort is required to understand how ecological networks influence range changes, especially when species’ interactions are antagonistic or mutualistic, or where they can be either, depending on abiotic circumstances [26]. More research also needs to consider the effect of species' interactions on fitness in natural populations beyond what can be deduced from population and metagenomics. The study of range changes would also benefit from more tests using experimental evolution, especially when combined with gene network studies, where functional genomes are known and frequencies of structural variants in chromosomes can be assessed. For example, studies could compare different forms of genomic organization, and ask how well these predict phenotypic trajectories. Such an approach was attempted in *Drosophila* studies many years ago (e.g. [114]), but can now be explored using resequencing to test how evolutionary responses relate to the distribution of positive and negative mutational effects (e.g. [115]).

How close are we to predicting maximum evolutionary rates in marginal populations in response to environmental change, beyond knowing that it must usually be increased in larger populations? We are confident that the advances presented in this volume move us much closer to such predictions, and to understanding how to maximize rates of evolution in communities already decimated by habitat and biodiversity loss, as they face the novel environments of the coming decades.

## Data accessibility

This article has no additional data.
